# Measurement of counter rotation of the innominate bones in the sagittal plane using a combination of rasterstereography of the back and frontal stereo-photogrammetry - a pilot study

**DOI:** 10.1186/1748-7161-9-S1-O2

**Published:** 2014-12-04

**Authors:** Silke Auler

**Affiliations:** 1 BUFA, Dortmund, Germany

## Introduction

Counter rotation of the innominate bones is an aspect of pelvic torsion composed of anterior rotation of one ilium and counter rotation of the contralateral ilium in the sagittal plane. It is regarded as an outcome parameter of conditions like leg length inequality, musculoskeletal dysfunction and spinal deviation. Assessment methods widely diversify in complexity [1]. Surface topography by video rasterstereography offers a reliable, contact free and radiation free method to assess the alignment of the pelvis from the posterior aspect [2]. Furthermore pelvic torsion is quantified as the solid angle between the surface normal to the dimples of venus providing a solid difference angle only without absolute figures on the orientation of the innominates.

## Objective

To investigate whether stereo-photogrammetric recording of anterior landmarks can be combined with simultaneous video rastersterography. Thus position data on anterior and posterior pelvic landmarks may be combined to receive figures of absolute rotational movement of the innominate bones in the sagittal plane.

## Methodology

In functional measurements with induced leg length discrepancy (LLD) 20 healthy subjects were examined with a hybrid device composed of video rasterstereography (Formetric III 4D®) and a frontal stereo-photogrammetric two-camera system to record markers indicating the left and right ASIS each. By computerised, mathematical consolidation the 3-D coordinates of all 4 pelvic landmarks were transformed into a common coordinate system allowing the calculation of spatial alignment of the innominates.

## Results

The distance between lumbar dimples was detected with a mean standard deviation of 1.38mm. The respective figure for the ASIS was 1.09mm. Anterior and posterior sagittal plane rotation of the innominate bones due to induced LLD was statistically significant (p<0.001). However, the correlation between LLD and rotation was weak (r<0.5). The mean excursion range was 5° respective 4° for the right and left innominate exhibiting counter rotation.

## Conclusion

The combination of surface topography and stereo-photogrammetry is applicable to assess and quantify the individual movements of left and right innominate bones in the sagittal plane separately. For a better understanding of inter individual differences in responding induced leg length inequality and to improve the clinical practicability further studies are recommended.

## References

[B1] Cooperstein R The relationship between pelvic torsion and anatomical leg length inequality: a review of the literature Journal of Chiropractic Medicine 2010 9 2 96 10.1016/j.jcm.2010.04.002 21629558 PMC2943661

[B2] Drerup B Hierholzer E 'Automatic localization of anatomical landmarks on the back surface and construction of a body-fixed coordinate system' Journal of biomechanics 1987 20 10 961 970 10.1016/0021-9290(87)90325-3 3693377

